# Sexual Aggression and Victimization Among Adolescents in School: Using a MixIRT Analysis to Examine Measurement Equivalence

**DOI:** 10.1002/ab.70022

**Published:** 2025-03-20

**Authors:** Thomas P. Gumpel, Anne Spigt

**Affiliations:** ^1^ The Hebrew University of Jerusalem Jerusalem Israel

**Keywords:** latent profile analysis, MixIRT, peer aggression, school aggression, sexual aggression, sexual victimization

## Abstract

Studies examining the frequency of sexual aggression and victimization in schools have compared different groups of respondents based on age, gender, or involvement in other types of school aggression. Between‐group comparisons assume measurement equality. We examine this assumption of measurement equality using a MixIRT analysis, which combines a latent profile analysis with a Rating Scale Model Item Response Theory analysis to determine whether sexual aggressors and victims can be divided into latent classes and whether the latent traits of sexual aggression or victimization have configural, metric and scalar equivalence and through an examination of differential item functioning (DIF). This is a secondary analysis of 3746 Israeli adolescents responding to a self‐report questionnaire regarding sexual aggression and victimization. Data analyses proceeded in five steps, and the unit of analysis was each respondent's responses to the aggressor and victim scales. We conducted a series of exploratory and confirmatory analyses of the aggression/victimization scale to examine configural equivalence, followed by a series of Latent Profile Analyses to determine metric and scalar equivalence. Finally, we examined DIF and Wright Maps using a Rating Scale IRT model. Four latent classes were identified. All items showed configural equivalence and most exhibited metric and scalar equivalence. An examination of DIF and Wright Maps showed that the structures of the latent traits for each latent class were fairly similar. However, for all latent classes, measures of sexual aggression and victimization failed to sample the full range of item difficulty (or endorseability).

Sexual aggression and bullying in schools are defined as the systematic use of physical or relational sexual aggression against a weaker peer (American Association of University Women 1993, 2001; Olweus 1993). In examining aggression and victimization, some researchers adhere to the participant‐role approach (Levy and Gumpel 2017; Salmivalli 2010), where participants are categorized into their primary behavioral roles based on established participant roles (for an extensive review, see Salmivalli 2010). However, these a priori classifications may be suspect based on recent qualitative observational studies of aggression and bullying based on long‐term observations of participants (Thornberg et al. 2013); for example, previous studies have found that participants engage in high levels of role‐switching (Gumpel et al. 2014; Huitsing and Veenstra 2012; Zioni‐Koren et al. 2022) and role‐overlapping (Jennings et al. 2010; Park and Cho 2021). In other words, most participants engage, to varying extents, in aggressor, assistant, bystander, help‐giver, outsider, and victim behaviors, where almost everyone is doing nearly everything, and so an a priori assignment to a fixed participant role may be contraindicated. A primary goal of the current research, therefore, is to examine an a‐theoretical typology of participation and to compare different groups of participants and their understanding of the latent traits of sexual aggression and victimization in schools.

Nylund et al. (2007) used Latent Class Analyses (LCA focuses on binary indicators, whereas LPA focuses on continuous indicators) to classify student victimization experiences. They found three specific patterns of responses anchored primarily in frequency rates: a victimized class, a sometimes‐victimized class, and a non‐victim class. Other studies have found similar results, with patterns of victimization characterized by highly victimized, moderately victimized, or uninvolved/low‐risk classes (Ashrafi et al. 2020; Bettencourt and Farrell 2013; Gage et al. 2014). Furthermore, in an examination of physical aggression, Wang et al. (2009) found that the different latent classes of respondents were differentially associated with various outcomes. More recently, Gumpel and Gotdiner (2023) used LPA procedures to examine the relationship between aggression, victimization, and social dominance orientation while examining both aggressors and victims. To our knowledge, these procedures have not been applied to analyzing sexual aggression or victimization. Sexual aggression and victimization in schools are widespread (Smith et al. 2020) and have been primarily studied by examining frequencies of perpetration and victimization; however, there appears to be scant research regarding the structure of the latent traits of sexual aggression and victimization. A primary goal of this research is to identify and examine potential respondent groups and response patterns.

## 1 Latent Groups and Latent Traits

Measurement equivalence is a central tenet in psychometrics (Rodriguez et al. 2023), and the lack of equivalence can prevent meaningful between‐group comparisons (Hambleton et al. 1991). On a scale level, researchers often use confirmatory factor analyses or structural equation modeling to examine scale‐level measurement equivalence. However, equivalence can also be examined on an item‐by‐item basis. Studies often fail to examine equivalence item level equivalence; instead, they assume a common latent trait with solely quantitative differences between respondent groups. Inherent in these procedures is the classical true score (Thorndike 1982) assumption that different types of respondents react to the measured latent trait similarly, essentially comparing “apples with apples and oranges with oranges.” Studies of measurement equivalence examine this assumption and can be meaningfully applied to self‐reports. Indeed, the subjective nature of self‐reports necessitates ensuring that different groups of respondents attach the same meaning or have the same concepts in mind and understand the concept along a similar scale with a common origin when responding to a specific item or set of items.

There is ample research that shows that direct and indirect aggression and victimization are positively correlated (Haltigan and Vaillancourt 2014). This victim‐offender overlap has been documented in the school aggression literature (DeCamp and Newby 2015; Semenza 2021); for example, Haltigan and Vaillancourt (2014) described these joint trajectories. The same appears to be true of sexual aggression and victimization (Jennings et al. 2010; Krahé and Berger 2020; Peterson et al. 2019). For example, Moyano et al. (2017) found that a powerful predictor of sexual aggression in a sample of adolescents was that the perpetrator was also a victim of sexual aggression.

Although critical for valid between‐group comparisons, the assumption of measurement equality, and hence the comparability of results, is often untested (Kankaraš et al. 2018; Lasker 2024). Tests of measurement equivalence examine three hierarchically linked forms of equivalence in which each level enables more robust between‐group comparisons. *Configural* or *structural* equivalence implies the similarity of data configurations or structures across groups. It is often measured by examining relationships between items (e.g., factor loadings) across groups, usually employing confirmatory factor analysis (CFA), which asks only if items are related to the same latent traits. *Metric (measurement unit) equivalence* examines the relationship between observed indicators and latent constructs, which is equal across groups, ensuring that items contribute similarly to the latent construct and allowing for valid comparisons of relationships involving those constructs. Metric equivalence implies the equality of measurement units across the latent trait across groups (i.e., the instrument measures the same latent construct for each group). Metric equivalence is a sufficient condition for comparing different scores across groups (Steenkamp and Baumgartner 1998); however, despite being based on equal measurement units of similar latent traits, they may not share the same origin on the scale (van de Vijver and Leung 2011), as some groups may be systematically higher or lower. As such, metric equivalence is insufficient for between‐group comparisons. *Scalar equivalence* goes further, testing whether item intercepts are invariant across groups, which is necessary for comparing latent means without bias from systematic differences (Steenkamp and Baumgartner 1998). While configural or structural equivalence is commonly evaluated in studies, metric and scalar equivalence receive less attention despite their importance for ensuring group comparisons (Cheung and Rensvold 2002; Meredith 1993). This oversight can lead to biased conclusions when latent constructs are not measured equivalently across groups (Putnick and Bornstein 2016).

Measurement equivalence is closely related to differential item functioning (DIF). DIF, or item bias, refers to differences in response probabilities between respondents with equal latent dispositions (Hambleton et al. 1991). This assumption can be examined via Item Response Theory (IRT) procedures and can be used to verify the equivalence or difference in groups’ understanding of the measured latent trait. For example, Rodriguez et al. (2023) examined DIF on a scale of parenting skills and found that for Black and Asian parents, two items had nonuniform DIF. However, to the best of our knowledge, this fundamental assumption has never been examined in the peer aggression and victimization literature, specifically in the domain of sexual aggression and victimization.

IRT models have recently been applied to understand whether groups identified in LPA procedures respond to similarly structured latent traits. This so‐called Mixture IRT (or MixIRT) model assumes that sub‐populations exist as a function of membership in different latent classes (Sen and Cohen 2019). A MixIRT analysis (Rost 1990, 1991) combines LPA and IRT's strengths to account for within‐group and between‐group differences. It addresses the heterogeneity in a population by extracting latent classes and examining their understanding of the latent trait. Unlike usual IRT models, MixIRT models do not assume a single homogenous population divided between latent classes (Sen and Cohen 2019) and have been used to identify alternative response patterns (Karadavut 2021), allowing researchers to investigate differences between response groups. Initially, LPA is used to identify subgroups of individuals based on their response patterns to observed variables and assumes that subgroup members have similar response patterns. Next, measurement equivalence is measured by determining whether items have configural, metric, and scalar equivalence based on fit statistics. Depending on whether items have scalar equivalence, the structure of the latent trait is examined using Wright maps. If item comparisons are valid, different subpopulations can be compared.

The purpose of this study, therefore, was to analyze sexual aggression and victimization using this MixIRT model. Specifically, we ask whether we can a‐theoretically identify latent classes (i.e., the LPA is exploratory rather than confirmatory) of sexual aggressors and victims beyond gender, age, and ethnicity. Next, we examine measurement invariance using a Rating Scale Model and identify potential differences in the structure of the latent traits based on DIF and Wright Map analyses.

## 2 Methods

### 2.1 Participants

This study presents a secondary analysis of data presented elsewhere (see Gumpel 2008), where data were collected as part of a large national sample of middle and high schools in Israel. Gumpel (2008) described that all respondents participated in a national anti‐bullying intervention conducted in Hebrew and Arabic throughout the country. Data were collected by the Israeli Ministry of Education's Chief Scientist Office and were approved by the Ministry's IRB. Active, informed consent was obtained via letters from each classroom homeroom teacher to parents. Respondents could opt out at any time. Questionnaires were completed in pencil/paper format, scanned using OCR software (Survey System, 2005), and were completely anonymous. In Israel, there is some confusion between ethnicity and native language, primarily regarding the nation's Palestinian minority (roughly 20% of the population). Typically, the two prominent ethnicities are referred to as Jews and Arabs. However, being a Jew denotes a religious affiliation, whereas being an Arab denotes ethnicity (i.e., Arabs can be Jewish, Muslim, or Christian); hence, when we refer to ethnicity/language, we are relating to the primary language spoken at home and the language of instruction. Israeli schools are separated by language of instruction (Hebrew or Arabic) and not by religion or ethnicity. Hence, in this paper, we define ethnicity primarily by language of instruction and refer to this variable as “Ethnicity/Language.”

### 2.2 Instrumentation

School Violence Inventory (Gumpel 2008) is a self‐report measure comprising 75 items in eight modules. The current study only used the last two modules, which examine the respondent's self‐reports of sexual aggression or victimization in the school context. All items can be found on the Open Science site (https://osf.io/gfnt5) and are based on a three‐point response scale (0 = never occurs to 2 = occurs frequently). As described in Gumpel (2008), items dealing with sexual aggression were taken from the AAUW studies (American Association of University Women 1993, 2001) with slight modifications as required by the IRB committee. As the AAUW studies deal only with sexual victimization, items were added to also reflect sexual aggression (these items were reworded to reflect aggression). The values of CFI, TLI, and RMSEA for the sexual aggression scales were low. Based on modification indices, the original scale was reduced to 11 items that strongly fit the model (CFI = 0.94, TLI = 0.92, RMSEA = 0.06) with a new internal consistency score of (McDonald's *ω* = 0.80). For the 11 items of the sexual victimization scale, the internal consistency (McDonald's *ω* = 0.80), and the confirmatory factor analyses showed that the data fit the model (CFI = 0.91, TLI = 0.89, RMSEA = 0.07). All scales are summarized in Table 1.

**Table 1:** Internal reliability (McDonald's *ω*) and CFA fit for the six subscales of the SVI.

Scale	# Items	*ω*	CFI	TLI	RMSEA
Sexual aggression	10	0.80	0.94	0.92	0.06
Sexual victimization	11	0.80	0.91	0.89	0.07

### 2.3 Data Analytic Procedure

Data analyses proceeded in five steps, and the unit of analysis was each respondent's responses to the aggressor and victim scales. Initially, to validate our instruments' reliability, validity, and hence configural equivalence, we conducted a series of exploratory and confirmatory analyses of the aggression/victimization scale using the McDonald's *ω* module in STATA (Shaw 2021). First, we divided our sample into two random and equal‐sized sub‐samples. An exploratory principal component factor analysis was conducted on the first sub‐sample, and a maximum likelihood CFA was conducted on the second sub‐sample. Second, we conducted regression analyses to determine whether age, gender, and ethnicity/language add to the prediction of both sexual aggression and sexual victimization. Third, we conducted a series of Latent Profile Analyses using LatentGold software (v. 6.0.0.22019, Vermunt and Magidson 2019) with the responses on the 11 sexual aggressors and the 11 sexual victim items used as the input variables. LPAs specified one to eight classes, and we accepted the latent class structure with the best model fit as indicated by the Bayesian information criteria (BIC), the Akaike information criteria (AIC), the log‐likelihood scores, and entropy *R*². Measurement invariance was evaluated using the syntax module in LatentGold and was conducted to examine configural, metric, and scalar equivalence. In the fourth step, to further validate group membership as determined by the LPA, we conducted a multinomial logistic regression to examine whether age level, gender, ethnicity/language could predict posterior class membership. In the fifth stage, we conducted an RSM analysis using Winsteps (Linacre 2023). We examined DIF, and Wright maps for the sexual aggression/victimization scale in addition to reviewing each class identified by the LPA.

### 2.4 Transparency and Openness

This manuscript has been prepared according to the ethical standards dictated in the Journal Article Reporting Standards (Kazak 2018) of the American Psychological Association. We have reported all necessary study information, including participant recruitment methods, data exclusions, and manipulations, and all approaches to the measurement of study variables. We report how we determined our sample size, all data exclusions (if any), all manipulations, and all measures in the study. Instrumentation, syntax, and data for this study are available from the Open Science Framework (DOI 10.17605/OSF.IO/UDH4Q) or the corresponding author.

## 3 Results

### 3.1 Missing Data

The proportion of missing data across the sexual aggressor and victim scales was very similar (4.29% and 4.59%). Little's MCAR test was nonsignificant (*χ* ^2^[2] = 0.60, *ns*), suggesting that the data were missing completely at random. There was one clear missing data pattern (2% of the sample), where participants were missing aggressor scores. Since the number of missing cases was small and MCAR, no further treatment of missingness was necessitated.

### 3.2 Normality and Covariance

Based on the Kolmogorov–Smirnov (K–S) test for normality for both the sexual aggressor and victim scales, both scales were not normally distributed (combined K–S = 0.36, *p* < 0.001 and 0.30, *p* < 0.001, respectively). Visual inspection of histograms and QQ plots for the two scales suggested that although data were not skewed (skewness = 0.45 and 0.44, respectively), they were slightly leptokurtic (kurtosis = 1.73 and 1.84, respectively). We attempted to correct this slight non‐normality based on the inverse density function; however, the correction had little substantial effect. Accordingly, we ignore this slight non‐normality based on the large sample size (*n* = 3746) (Schmidt and Finan 2018). As IRT analyses are based on a unidimensional analysis of items, we conducted a CFA to establish that the two dimensions of sexual aggression could be combined into one factor: sexual aggression. The CFA analysis based on the two exogenous variables of sexual aggression and sexual victimization showed that the two subscales are part of the same latent trait (CFI = 0.98, TLI = 0.98, RMSEA = 0.04).

## 4 Descriptive Statistics

Completed questionnaires were received from 3476 students in 30 Israeli middle and high schools in Israel. All respondents were enrolled in the general education system. The sample population included students from the seventh to the twelfth grade (ages 12–18) as this group participated in the Ministry of Education's anti‐bullying program. All data were collected before the beginning of the program implementation in each school. Gender distribution was typically distributed (48.53% boys vs. 51.47% girls). Most respondents came from Hebrew language schools (84.8%). The two subscales were also correlated, *r* = 0.92, *p* < 0.001. Both subscales were significantly correlated with one another, means, standard deviations, and the correlation coefficients are presented in Table 2, broken down by gender.

**Table 2:** Means, standard deviations, and correlation coefficients for each aggression/victimization scale by gender. Male scale correlations are on the bottom left of the correlation matrix, and females are on the top right.

Females (*n* = 5875)	Males (*n* = 5480)		1	2
Mean (SD)	Mean (SD)			
0.65 (0.47)	0.67 (0.49)	1. Aggressor—sexual		0.93*
0.72 (0.49)	0.68 (0.48)	2. Victim—sexual	0.92*	

* *p* < 0.001.

## 5 LPA Analyses

An LPA based on sexual aggression and victimization using the biased‐adjusted three‐step approach (Bakk et al. 2016) includes three analytical steps. In the first step, latent clustering models are built based on a set of observed variables, and the most suitable model is selected. Respondents are classified into classes based on posterior probabilities in the second step. Class identification is verified via multinomial logistic regression based on these posterior probabilities. The third step examined the relationship between the classes and the SVI's direct and indirect aggression scales.

**Figure 1:**
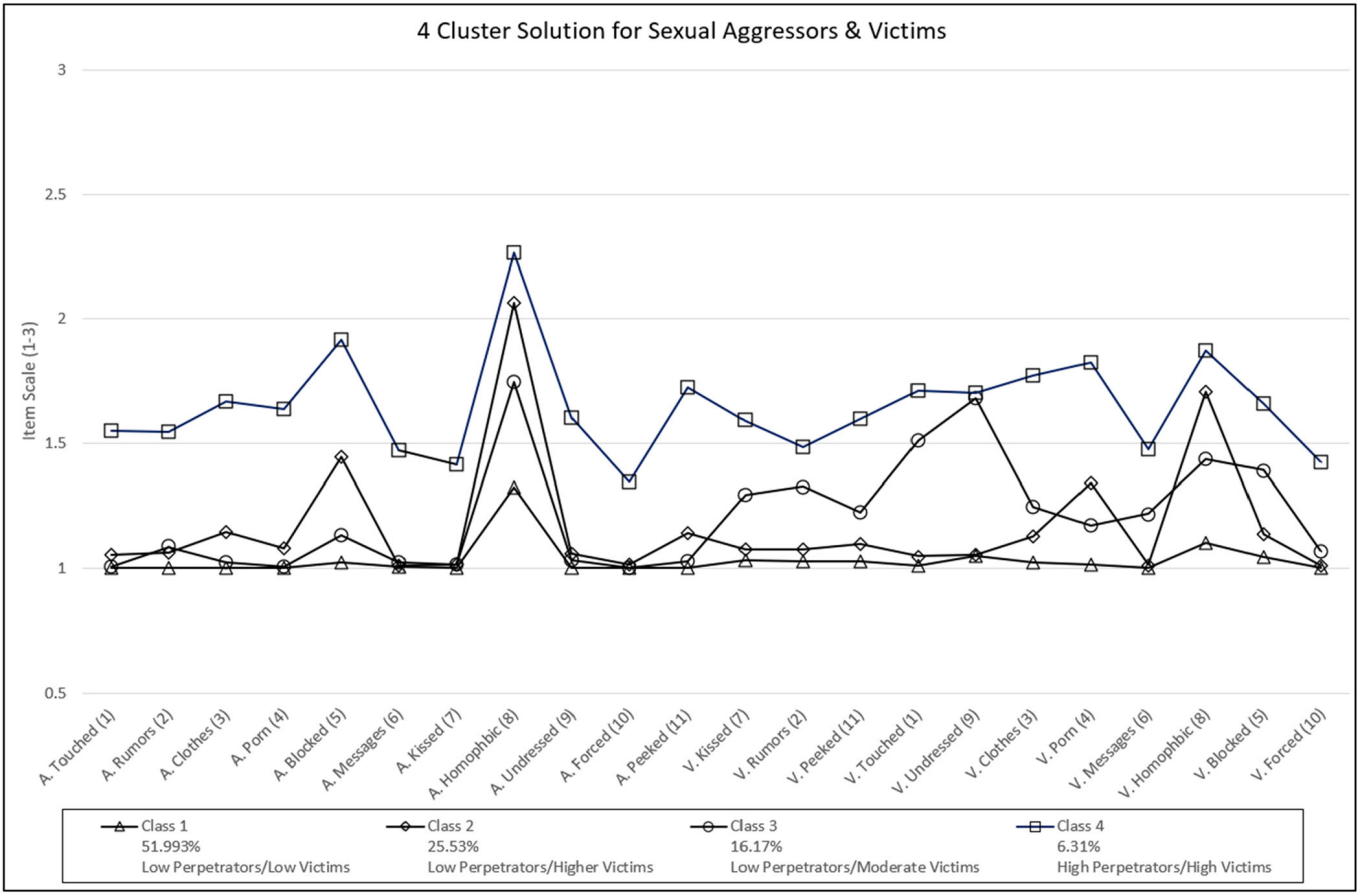
Class solutions. A, aggressor; V, victim. Numbers in parentheses are item numbers (see Appendix). [Color figure can be viewed at wileyonlinelibrary.com]

Table 3 shows the latent profile information based on the log‐likelihood (LL), the BIC and AIC, the number of parameters (NPAR) of criteria, the Vuong–Lo–Mendell–Rubin test which examines the probability that a *k*‐class model fits better than a *k* − 1 class model, and entropy *R* ^2^ (a measure of the separation between classes). BIC and AIC are based on the log‐likelihood of a given model, each applying a different penalty for model complexity (Finch 2015); the smaller the value, while considering degrees of freedom, the better fitting the model. After calculating fit statistics for eight potential latent profiles, the four‐cluster model was selected as the best fit for the data. Even though BIC and AIC are smaller when classified into more groups, examining the sample proportion in each class showed marginal probabilities of less than five percent for any class solution with greater than four classes. Considering the sample size of 3376 (using listwise deletion), the typical rule of thumb is that classes should be no smaller than approximately 5% (Muthén and Muthén 2000). In addition, we used G*Power (version 3.1.9.6, Erdfelder et al. 1996) for a post hoc examination of power for an analysis of variance; a class with 4% of respondents would yield 96% power; thus, any solution with a greater than four‐cluster solution was counter‐indicated. As is evident, for both scales, the four groups differ primarily in frequency rather than structure (see Figure 1). This effect is known as the “Salsa Effect” (Sinha et al. 2021), where the indicators of the identified classes run parallel to one another, suggesting that they are representative of scale severity. Accordingly, we would classify these four groups as Class 1 as low perpetrators and low victims, Class 2 as low perpetrators and slightly higher victims, Class 3 as low perpetrators and moderate victims, and Class 4 as high perpetrators and high victims. However, in referring to Figure 1, it is clear that, generally, scores for all four groups appear to be low (i.e., on a three‐point scale); only Class 4 consistently receives scores above the median score of 1.5.

**Table 3:** Bayesian information criterion (BIC), Akaike's information criterion (AIC), Vuong–Lo–Mendell–Rubin (VLMR), *p* VLMR, and entropy *R* ^2^ for Classes 1–8, with three inactive covariates (grade‐level, gender, language), *N* = 3376.

Classes	LL	BIC	AIC	NPAR	VLMR^a^	Entropy *R* ^2^
1	−21851.07	44050.52	43790.14	44		1
2	−19448.82	39451.89	39037.64	70	4804.50	0.83
3	−18869.03	38498.18	37930.06	96	1159.58	0.74
4	−18387.99	37741.96	37019.98	122	962.08	0.77
5	−18159.72	37491.29	36615.44	148	456.54	0.76
6	−17984.08	37345.88	36316.16	174	351.28	0.76
7	−17849.45	37282.49	36098.91	200	269.26	0.75
8	−17772.86	37335.16	35997.72	226	153.19	0.75

*Note:* Vuong–Lo–Mendell–Rubin adjusted likelihood test. Rectangle denotes the chosen model.

Abbreviations: AIC, Akaike information criterion; BIC, Bayesian information criterion; LL, log likelihood (model); NPAR, number of parameters.

^a^ VLMR indices significant at *p* < 0.001.

To further evaluate the four‐cluster solution, we conducted multinomial regressions using the posterior class as the base outcome to determine whether the grade, gender, ethnicity/language, and sexual aggression or victimization could predict the cluster membership. The predictor variables were able to predict posterior class membership, *χ* ^2^ (10,438) = 19276.67, *p* < 0.001, Pseudo *R* ^2^ = 0.92, and *χ* ^2^ (10,438) = 20774.15, *p* < 0.001, Pseudo *R* ^2^ = 0.93, respectively.

We examined class membership and the distal variables based on the bias‐adjusted third step, including the distal variable relationship to sexual aggression. Grade (Wald = 36.67), gender (Wald = 303.62), ethnicity/language (Wald = 373.11), and victim sexual (Wald = 263.92) were significantly different at the *p* < 0.001 level. The model for the distal variables' relationship to sexual victimization classes was significant (Wald = 377.91, *p* < 0.001). Grade (Wald = 18.20), gender (Wald = 117.29), language (Wald = 373.11), and aggressor sexual (Wald = 298.21) were significantly different at the *p* < 0.001 level.

## 6 MixIRT and Measurement Equivalence

Measurement equivalence was examined via the syntax module of LatentGold (Kankaraš et al. 2018; Vermunt and Magidson 2019). An LCA establishes measurement equivalence when class‐specific conditional response probabilities are equal across groups (Kankaraš et al. 2018). This assumption can be tested by restricting specific sets of model parameters across groups and determining the model fit and the existence of DIF, using the BIC and AIC information criteria as they correct for the problem of large sample size that can artificially inflate likelihood ratio statistic and its use in comparison of model fits. We also examined the value of the L2 difference statistic, calculated per parameter, to estimate the actual change in fit better while considering model parsimony.

Table 4 shows that all items aside from item 15 (victim—“shown porn”) achieve metric equivalence. As is evident in the size of the BIC statistic, the BIC for all items is smaller than the basic heterogeneous model. This finding is further reinforced by comparing differences in the L2 parameters (items with an L2 ≥ ≈ 5 are suspect); in other words, the instrument measures the same latent construct for each latent group. Next, we examine scalar equivalence (partial DIF) by comparing each item with the partial model as the basis for examining scalar equivalence. These results are shown in Table 5 and show that for item 8 (aggressor—“homophobic comments”) and item 19 (victim—“homophobic comments,” there is no scalar equivalence (i.e., there is no measurement equivalence as both groups do not have the same origin on these two items).

**Table 4:** Comparison of heterogenous model for scalar and metric DIF for each item's Bayes information criteria (BIC) for aggressors (A) and victims (V), *N* = 2746.

		LL	BIC (LL)	AIC (LL)	NPAR	*L*²	*df* a	Difference in *L* ^2^ per parameter
	Heterogeneous model	−15805.5433	33733.0840	32147.0867	268	16808.5385	2478	
	(Scalar and metric DIF)							
1.	A. Touched—metric DIF	−15812.9298	33724.1032	32155.8596	265	16823.3114	2481	−2.46
2.	A. Rumors—metric DIF	−15807.3976	33713.0388	32144.7951	265	16812.2470	2481	−0.62
2.	A. Clothes—metric DIF	−15807.2859	33712.8154	32144.5718	265	16812.0236	2481	−0.58
4.	A. Porn—metric DIF	−15808.6047	33715.4530	32147.2093	265	16814.6611	2481	−1.02
5.	A. Blocked—metric DIF	−15805.5538	33709.3512	32141.1075	265	16808.5593	2481	0.00
6.	A. Messages— metric DIF	−15808.0171	33714.2779	32146.0343	265	16813.4861	2481	−0.82
7.	A. Kissed (and—metric DIF	−15805.8084	33709.8605	32141.6169	265	16809.0687	2481	−0.09
8.	A. Homophobic— metric DIF	−15815.9179	33730.0795	32161.8358	265	16829.2876	2481	−3.46
9.	A. Undressed— metric DIF	−15805.9797	33710.2031	32141.9594	265	16809.4112	2481	−0.15
10.	A. Forced—metric DIF	−15806.1832	33710.6100	32142.3663	265	16809.8182	2481	−0.21
11.	A. Peeked—metric DIF	−15807.8796	33714.0029	32145.7593	265	16813.2111	2841	−0.78
12.	V. Touched—metric DIF	−15805.9627	33710.1690	32141.9253	265	16809.3772	2481	−0.14
13.	V. Rumors—metric DIF	−15809.2784	33716.8004	32148.5568	265	16816.0086	2481	−1.25
14.	V. Clothes—metric DIF	−15807.2590	33712.7617	32144.5181	265	16811.9699	2481	−0.57
15.	V. Porn—metric DIF	−15829.9124	33758.0684	32189.8247	265	16857.2765	2481	−8.12
16.	V. Blocked—metric DIF	−15806.1809	33710.6054	32142.3618	265	16809.8136	2481	−0.21
17.	V. Messages— metric DIF	−15809.5441	33717.3319	32149.0883	265	16816.5401	2481	−1.33
18.	V. Kissed—metric DIF	−15810.7591	33719.7618	32151.5181	265	16818.9699	2481	−1.74
19.	V. Homophobic— metric DIF	−15809.9604	33718.1644	32149.9208	265	16817.3726	2481	−1.47
20.	V. Undressed— metric DIF	−15807.0320	33712.3077	32144.0640	265	16811.5158	2481	−0.50
21.	V. Forced—metric DIF	−15809.3848	33717.0132	32148.7696	265	16816.2214	2481	−1.28
22.	V. Peeked—metric DIF	−15806.8768	33711.9972	32143.7536	265	16811.2054	2481	−0.44

*Note:* Rectangle denotes an item without metric equivalence.

Abbreviations: AIC, Akaike information criterion; BIC, Bayes Information Criteria; LL, log likelihood; Npar, number of parameters.

^a^ All comparisons are significant, *p* < 0.001.

**Table 5:** Comparison of partial DIF (metric equivalence) for each item's Bayes information criteria (BIC) for aggressors (A) and victims (V), *N* = 2746.

		LL	BIC (LL)	AIC (LL)	Npar	*L*²	*df* a	Difference in *L* ^2^ per parameter
	Partial DIF	−15922.0558	33443.5276	32248.1116	202	17041.5635	2544	
	(metric equivalence)							
1.	A. Touched—scalar DIF	−15926.3235	33396.6377	32242.6471	195	17050.0989	2551	−0.61
2.	A. Rumors—scalar DIF	−15923.2936	33390.5779	32236.5873	195	17044.0391	2551	−0.18
3.	A. Clothes—scalar DIF	−15923.0989	33390.1884	32236.1978	195	17043.6496	2551	−0.15
4.	A. Porn—scalar DIF	−15922.5377	33389.0660	32235.0754	195	17042.5272	2551	−0.07
5.	A. Blocked—scalar DIF	−15927.8264	33399.6434	32245.6528	195	17053.1046	2551	−0.82
6.	A. Messages— scalar DIF	−15923.9272	33391.8450	32237.8543	195	17045.3062	2551	−0.27
7.	A. Kissed—scalar DIF	−15924.5084	33393.0074	32239.0168	195	17046.4686	2551	−0.35
8.	A. Homophobic— scalar DIF	−16077.5785	33699.1477	32545.1570	195	17352.6089	2551	−22.22
9.	A. Undressed— scalar DIF	−15928.7621	33401.5148	32247.5242	195	17054.9760	2551	−0.96
10.	A. Forced—scalar DIF	−15922.1469	33388.2843	32234.2937	195	17041.7455	2551	−0.01
11.	A. Peeked—scalar DIF	−15924.4399	33392.8705	32238.8799	195	17046.3317	2551	−0.34
12.	V. Touched—scalar DIF	−15930.3298	33404.6503	32250.6597	195	17058.1115	2551	−1.18
13.	V. Rumors—scalar DIF	−15925.3919	33394.7745	32240.7839	195	17048.2357	2551	−0.48
14.	V. Clothes—scalar DIF	−15925.6718	33395.3341	32241.3435	195	17048.7953	2551	−0.52
15.	V. Porn—scalar DIF	−15930.7473	33405.4851	32251.4945	195	17058.9463	2551	−1.24
16.	V. Blocked—scalar DIF	−15933.4463	33410.8832	32256.8926	195	17064.3444	2551	−1.63
17.	V. Hit On—scalar DIF	−15936.7448	33417.4802	32263.4896	195	17070.9414	2551	−2.10
18.	V. Kissed—scalar DIF	−15929.7644	33403.5195	32249.5289	195	17056.9807	2551	−1.10
19.	V. Homophobic— scalar DIF	−15959.1608	33462.3122	32308.3216	195	17115.7734	2551	−5.30
20.	V. Undressed— scalar DIF	−15917.3629	33378.7164	32224.7258	195	17032.1776	2551	0.67
21.	V. Forced—scalar DIF	−15922.9634	33389.9174	32235.9268	195	17043.3786	2551	−0.13
22.	V. Peeked—scalar DIF	−15924.5866	33393.1638	32239.1732	195	17046.6250	2551	−0.36

*Note:* Rectangle denotes an item without metric equivalence.

Abbreviations: AIC, Akaike information criterion; BIC, Bayes Information Criteria; LL, log Likelihood; Npar, number of parameters.

^a^ All comparisons are significant, *p* < 0.001.

## 7 MixIRT Analyses

Item analysis involves an examination of fit statistics for each item in the sexual aggressor and victimization scales, providing the researcher with an indication of how well the respondent's actual item response pattern matched their expected item response pattern. All IRT analyses were conducted with WINSTEPS (v. 5.4.2.0, Linacre 2023). Two indices are used: the *infit mean square* (*mnsq*) and the *infit t* (Wright and Masters 1982; Wright & Stone 1979). The infit mean square is considered an indicator of the effect size of the misfit. In contrast, the standardized *z* score (zstd) is considered an indicator of the statistical significance of that effect with a theoretical mean of “0” and variance of “1.” These statistics can be applied to either persons or items (summed as a scale or individually). Items are considered misfitting if the effect is also statistically significant: Overall *infit t* is expected to have a mean of “0” and a standard deviation of “1” (Adams and Khoo 1996). The item *infit mnsq* in this scale is 1.09 and is nonsignificant (*zstd* = 1.54). Likewise, the *outfit mnsq* for the scale is 0.85 and is also nonsignificant (*zstd* = −1.10). Item separation evaluates the quality of test items, identifies items contributing most to the precision of the measurement instrument, and assists in gauging the overall reliability and validity of the instrument. Item separation values above 2.0 are considered acceptable, indicating moderate to high reliability in item difficulty estimates; for this scale, item separation = 14.01, indicating high reliability in item difficulty estimates. Person separation values above 2.0 are also considered acceptable, suggesting that the test can effectively differentiate between individuals with different trait levels. In the current case, person separation = 0.76, indicating that the instrument cannot adequately distinguish between respondents.

**Figure 2:**
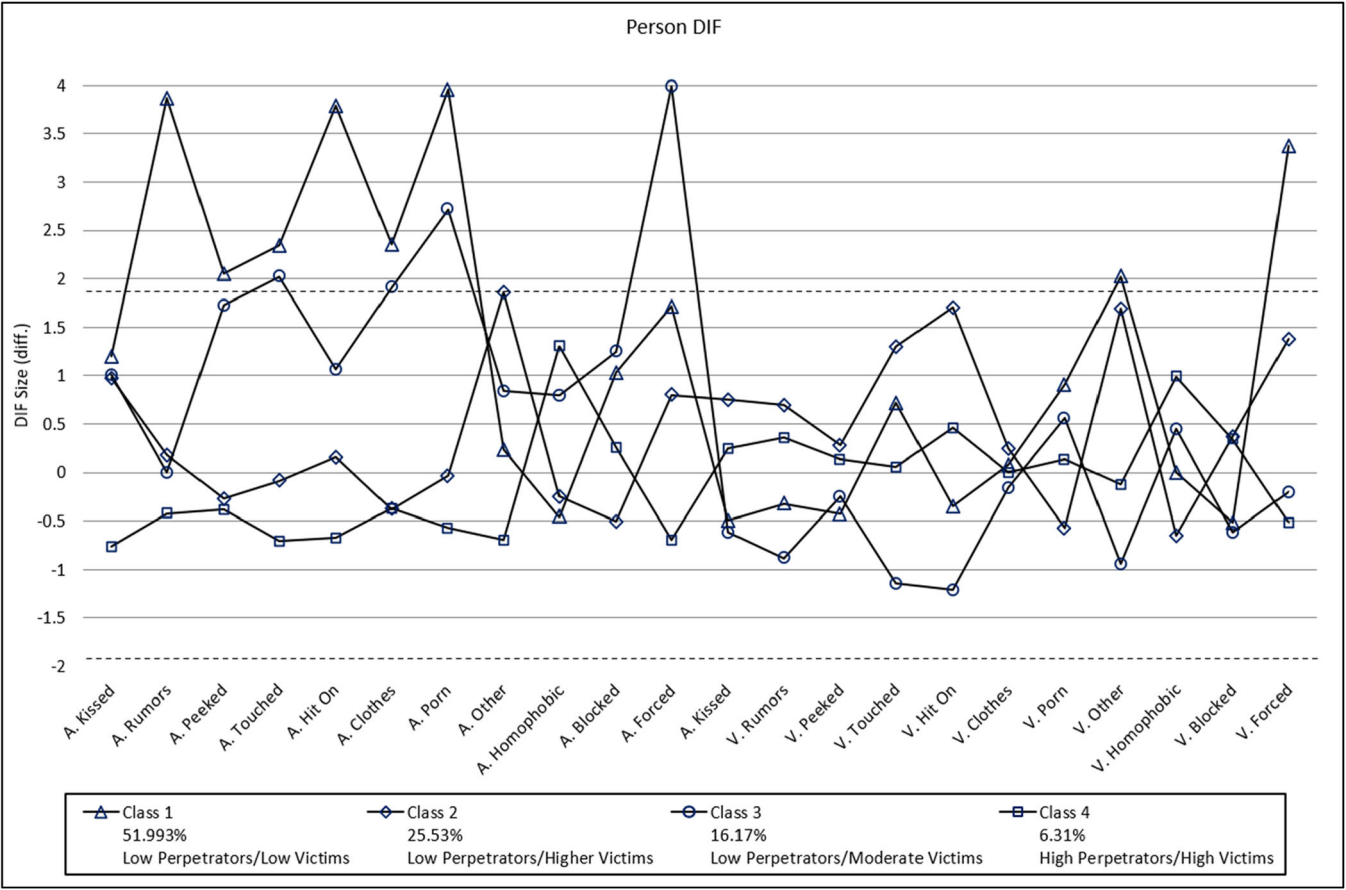
DIF by latent response class. Horizontal dashed lines mark upper and lower boundaries of acceptable DIF, no DIF if *t* > |1.96 |. A, aggressor; V, victim. Numbers in parentheses are item numbers (see Appendix). [Color figure can be viewed at wileyonlinelibrary.com]

Figure 2 shows DIF analyses for both subscales by latent class. The horizontal and parallel dashed lines demarcate acceptable DIF ranges based on Student's *t* scores between ± 1.96 (Zwick et al. 1999). Examination of the Person DIF chart shows that although the four classes have responded differently, they generally have low scores on both the aggressor and the victim scales. Most of the DIF scores fall above or below the two horizontal lines. There appears to be more DIF for the second class for the aggression items and the fourth class for the victimization items.

**Figure 3:**
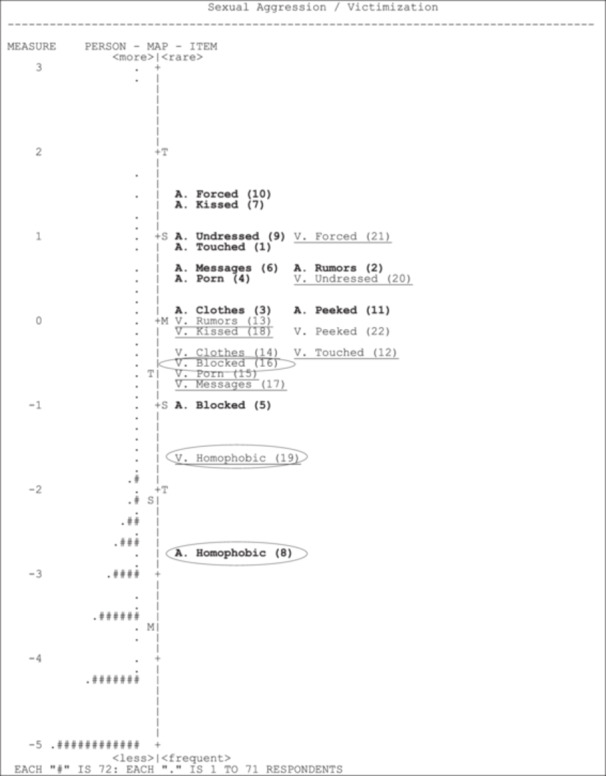
Wright Map for sexual aggression/victimization scales, A, aggressor; V, victim, *N* = 3376. Aggression items are bolded, and victim items are underlined to facilitate reading. Circled items have problematic measurement equivalence.

Despite this amount of significant DIF scores, an examination of the item Wright Maps (see Figure 3, Wilson 2005) shows that, for the most part, the basic structures of the latent trait for aggressors and victims are similar. Noticeable, however, are the aggressor and victim items relating to using or being exposed to homophobic comments. These are also the two items without scalar equivalence. This graphical representation compares the relative endorseability of the items (item difficulty) and respondent distribution. Circled items lack metric (item 15: V‐Porn) or scalar (items 8 and 19: “homophobic”) equivalence. Examining the items' relative fit allows for identifying the sensitivity of items and whether they cover the entire spectrum of possible difficulty. Likewise, examining the spread of respondents allows for reviewing items that are too easy or difficult to endorse despite having an adequate item separation score.

The left pane of Figure 3 shows that the majority of respondents are centered a bit above the mean (each “#” denotes 72 respondents, and each “.” denotes 1–71 respondents) at a logit score of “0”. Also evident is that for many respondents, each scale item is un‐endorsable (i.e., many respondents appear lower in the Person Map than the easiest items to endorse, as either aggressor or victim). Examination of the items on the right side of this panel shows the spread of items. Items 8 and 19 (aggressor or victim “homophobic”) are the easiest items to endorse, and item 10 (aggressor “forced”) is the most difficult to endorse. Most items are clustered between the mean item difficulty and one standard deviation above it. It is clear from Figure 3 that the instrument is not sampling the range of possible sexual aggression acts, particularly in the low range.

## 8 Discussion

The primary goal of this study was to conduct a MixIRT analysis of the sexual aggression and victimization scales to determine if latent response classes exist and whether there is measurement equivalence across latent classes. Based on the LPA component of the MixIRT analysis, it is possible to disaggregate sexual aggression and victimization scores by levels or frequency of involvement levels. Different classes respond to the latent traits in similar manners and engage in high frequencies of both sexual aggressor and victimization behaviors. It is important to remember that these data are from self‐reports. As such, they should not be seen as indicators of what the respondents did or how they behaved but instead what they think they did. This is an inherent problem with self‐reports of antisocial behavior or victimization.

Most interesting from the LPA is that there is no fifth group, that is, no respondent class of aggressors who are not victims. These findings are in contrast to an a priori imposed group structure in that other research has identified “pure aggressor” (Gumpel and Sutherland 2010; Schwartz 2000). Two primary taxonomies are most often cited regarding participant roles. In the first approach, respondents are divided by age and gender standard score cut‐offs into four respondent groups (pure aggressors, pure victims, aggressive victims, and uninvolved). They are primarily rooted in Olweus's early work (1978, 1993), and the second approach, based on Salmivalli's research (Salmivalli et al. 1996), participants are divided into between six and eight participant roles (Salmivalli 1999, 2010). Both approaches describe the aggressor who is not also a victim (the pure aggressor in the former and or the “ringleader” in the latter).

Our findings challenge this assertion, at least regarding sexual aggression/victimization, and show that all aggressors are also victims and are aggressor‐victims (Ashrafi et al. 2020; Bettencourt and Farrell 2013; Gage et al. 2014; Imuta et al. 2022; Nylund et al. 2007). In support of previous findings regarding sexual aggression and victimization in adults, the amount of role‐overlap was extreme: we found no sexual aggressors who were not also victims. These results partially confirm previous research on sexual aggression in schools. For example, Fineran and Bolen (2006) found that about half of the girls who were sexually harassed were also sexual aggressors. A more precise understanding of the structure of these roles and role‐switching between them should be further examined using observational studies, as positivistic one‐off questionnaires fail to consider the fluidity of changing roles and targets. For instance, we cannot tell who the aggressive victim targets: Does s/he target and retaliate against their aggressor? Or perhaps someone lower on the social status scale? Do they repeatedly target the same victim? Much is still to be learned about how and why participants change roles and how they see their role‐switching. Future research should address these issues.

DIF analyses are instrumental in establishing metric and scalar equivalence. In the current study, the “homophobic” items failed to achieve metric or scalar equivalence. Indeed, these items have little discriminatory value based on the LPA and the Wright Map. One possible (and worrying) explanation is that homophobic aggression and victimization are so common that respondents did not see them as extreme behaviors but as part of their ambient culture (Albaladejo‐Blázquez et al. 2019; Tucker et al. 2016). Nevertheless, future research should further explore these issues to determine why these different respondent groups did not achieve scalar equivalence. Rasch modeling helps to identify problematic items and assesses whether the latent trait measures the intended construct with equal sensitivity and across the full range of potential behaviors. For both aggression and victimization, direct aggression items (i.e., forcing or being forced into an unsolicited sexual act, kissing or being kissed uninvited, touching or being touched) are at the more severe end of the latent trait for both sexual aggressors and victims. This research questions the validity of these two approaches. It suggests that a more parsimonious approach may rely solely on frequencies of behaviors rather than theoretically determined and presumed stable participant roles. This is not surprising, and there are parallels between direct physical and indirect aggression.

The IRT analysis suggests that this measure of sexual aggression and victimization is not adequately measuring the breadth of the aggression/victimization spectrum. It is important to note that issues of sexual aggression have changed considerably since the AAUW first published its survey in 1993 (American Association of University Women 1993), specifically since the advent of the #METOO movement (Acquaviva et al. 2021; Daigle 2021). Furthermore, definitions of sexual identity have changed considerably over the last decade. For example, a quarter of GEN Z'ers expect to identify their gender differently from their cisgender at least once during their lifetime (Luttrell and McGrath 2021). With increasing global awareness of gender diversity, ideas about gender are changing. Visibility of trans people, genderqueer, agender, and otherwise gender fluid identification is becoming more common (Minter 2012). For example, in Israel, Jacobson and Joel (2018) showed that cis‐children, not gender minorities, do not describe their gender experience as binary; these changes also apply to schools (Giovanardi et al. 2019). This shift emphasizes the need for a reconceptualization of sexual aggression and victimization; future research should address these issues.

Our study makes a unique contribution to the aggression research by combining an LPA and Rasch analysis within a MixIRT methodology to examine measurement equivalence between groups. We were able to show configural, measurement, and scalar equivalence as a necessary prerequisite for examining both measurement scales and the items comprising those scales. We were able to prove that measures are, indeed, measuring the same constructs allowing for between‐group comparisons. To our knowledge, this approach is the first application in the aggression/victimization literature. By combining LPA and IRT analyses, we gained a more comprehensive understanding of subgroups of aggressors and victims. Currently, there is no simple way to conduct these analyses. We used two different data analysis programs (LatentGold andWinsteps, Linacre 2023; Vermunt and Magidson 2019); hopefully, more efficient software will become available.

## Author Contributions

Thomas P. Gumpel served as lead for conceptualization, data curation, formal analysis, project administration, methodology, writing – review editing, and supervision. Anna Spigt served in a supporting role in data curation, formal analysis, and writing original drafts.

## Conflicts of Interest

The authors declare no conflicts of interest.

## Data Availability

The data supporting this study's findings are available in the Open Science Framework and can be accessed at: https://doi.org/10.17605/OSF.IO/UDH4Q.

## 1

Sexual aggression/victimization items

All items appear twice, worded for an aggressor and worded for a victim.

1. Touched, grabbed, or pinched in a sexual way
2. Had sexual rumors spread about them
3. Had clothing pulled at in a sexual way
4. Shown, given, or left sexual pictures, photographs, illustrations, messages, or notes
5. Had their way blocked in a sexual way
6. Had sexual messages/graffiti written about them on bathroom walls, in locker rooms, etc.
7. Forced to kiss someone
8. Called Gay or Lesbian
9. Had clothing pulled off or down
10. Forced to do something sexual other than kissing
11. Spied on while dressing or showering

Adapted from: American Association of University Women (1993).
